# Evidence of an Off-Resonant Electronic Transport Mechanism in Helicenes

**DOI:** 10.1021/acs.jpclett.4c01425

**Published:** 2024-08-07

**Authors:** T. de Ara, C. Hsu, A. Martinez-Garcia, B. C. Baciu, P. J. Bronk, L. Ornago, S. van der Poel, E. B. Lombardi, A. Guijarro, C. Sabater, C. Untiedt, H. S. J. van der Zant

**Affiliations:** † Departamento de Física Aplicada and Instituto Universitario de Materiales de Alicante (IUMA), Universidad de Alicante, Campus de San Vicente del Raspeig, E-03690 Alicante, Spain; ‡ Department of Quantum Nanoscience, Delft University of Technology, Delft 2628CJ, The Netherlands; ¶ Departamento de Química Orgánica and Instituto Universitario de Síntesis Orgánica, Universidad de Alicante, Campus de San Vicente del Raspeig, E-03690 Alicante, Spain; § Department of Physics, Florida Science Campus, University of South Africa, Florida Park, Johannesburg 1710, South Africa

## Abstract

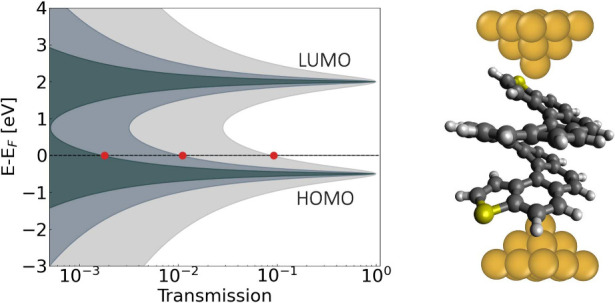

Helical molecules have been proposed as candidates for producing spin-polarized currents, even at room conditions, due to their chiral asymmetry. However, describing their transport mechanism in single molecular junctions is not straightforward. In this work, we show the synthesis of two novel kinds of dithia[11]helicenes to study their electronic transport in break junctions among a series of three helical molecules: dithia[*n*]helicenes, with *n* = 7, 9, and 11 molecular units. Our experimental measurements and clustering-based analysis demonstrate low conductance values that remain similar across different applied voltages and molecules. Additionally, we assess the length dependence of the conductance for each helicene, revealing an exponential decay characteristic of off-resonant transport. This behavior is primarily attributed to the misalignment between the energy levels of the molecule–electrodes system. The length dependence trend described above is supported by *ab initio* calculations, further confirming an off-resonant transport mechanism.

The study of electronic transport properties in single molecules has garnered significant attention due to their versatile and programmable structural features. This interest has driven advancements in molecular-scale devices, with the goal of harnessing the unique chemical and physical properties of individual molecules.^1,2^ A topic that has gained attention in recent years is the research on chiral molecules as spin valve-type molecules. Chirality is a fundamental symmetry property ubiquitous in nature and found in DNA, amino acids, and sugars. In this regard, due to their chirality, helical molecules have been identified as potential candidates for electrons to become spin-polarized after being transmitted through these molecules.^3^ By using their symmetry properties, these chiral conformations would discriminate spin currents without relying on ferromagnetic electrodes or applied magnetic fields. For example, spin polarization of electrons has been demonstrated using nonpolarized light through a chiral molecular structure such as DNA.^4^ These measurements show that charge and spin transport are coupled, a phenomenon that is called chirality-induced spin selectivity (CISS).^3,5^

To understand the CISS phenomenon, it is fundamental to study single-molecule junctions, where electronic transport occurs out of equilibrium and is typically driven by an external bias voltage that induces a difference in the chemical potentials across the metallic leads. While considering this scenario, discrepancies have been reported between electronic transport experiments and theoretical calculations.^6−8^ In this context, chiral structures such as helicenes have been proposed^9−14^ due to their helical configuration and the possibility to isolate their mirror images, labeled as enantiomers. However, before proceeding with the detection of spin currents, it is important to address first their charge transport in single molecule-metal junctions and determine if they are good candidates, which requires a basic understanding of their conductance and the anchoring to the electrodes.

Throughout this work, electron transport is investigated for a series of chiral molecules at ambient conditions using the mechanically controllable break junction (MCBJ) technique. Our approach involves the study of a set of helicenes with a helix structure and anchoring links incorporated to the molecular structure to enhance conductivity^15,16^ (schematic illustrations are in Figure 1(a)). Henceforth, we focus on studying dithia[n]helicenes, where n represents the number of aromatic rings, specifically n = 7, 9, and 11. By analyzing these helicenes of varying sizes, we aim to gain insights of the nature of electronic transport mechanism in this system from the relationship between molecular length and electron transport characteristics.

**Figure 1:**
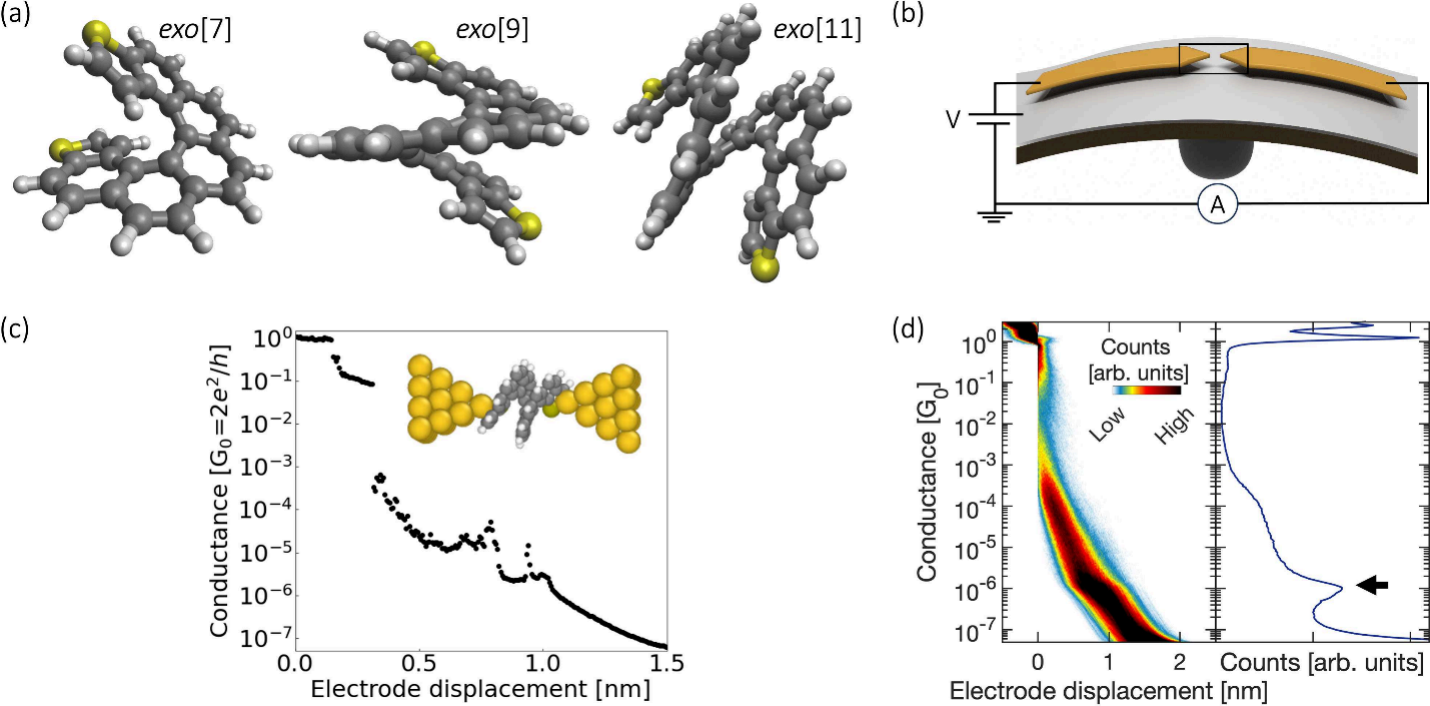
MCBJ measurement methodology and molecular schemes. (a) Chemical structures of the dithia[*n*]helicenes: exo[7], exo[9], and exo[11]. Yellow, gray, and white spheres represent sulfur, carbon, and hydrogen atoms, respectively. (b) Schematic representation of the MCBJ setup. Two gold electrodes with a notch are displayed. When the rod is pushed along the vertical direction, the junction stretches and a molecular conductor can be established. (c) Example of a breaking trace showing conductance in units of G_0_ as a function of the relative displacement measured at a fixed bias voltage of 0.1 V. Inset displays the exo[11]dithiahelicene candidate bridging the gold electrodes. (d) 2D and 1D histograms of the raw data containing ten thousand consecutive breaking traces obtained for exo[11]dithiahelicene at 0.1 V. The black arrow points to the peak due to an amplifier artifact.

In our molecules sulfur atoms are incorporated into the molecular structure using thiophene rings. In such a way, anchoring groups such as thiols^17−23^ are removed. This design choice originates from the idea of reducing potential barriers when the molecule bridges between electrodes while maintaining the conjugation throughout the molecule.^24^ This concept was supported by preliminary Density Functional Theory (DFT) calculations on isolated molecules, revealing the development of molecular orbitals that include the contribution of the sulfur atoms. Additionally, sulfur atoms are expected to provide mechanical stability while the helical structure offers its inherent flexibility.^25^ Furthermore, we explore the influence of the sulfur atoms by arranging the sulfurs in two different positions. First, the sulfurs located by facing outside the helical axis as illustrated in Figure 1(a) and second, the sulfurs positioned on the opposite side of the thiophene, facing the helical axis. We refer to these configurations as *exo* and *endo*, respectively.

In our previous works,^15,16^ we described the preparation of both configurations for [7] and [9] dithiahelicene. The preparation of the yet unreported exo[11] and endo[11] dithiahelicene was achieved by adapting these modular syntheses to a larger central phenanthrene fragment. This process involves our state of the art methods of Pd-catalyzed coupling reactions followed by a LED-driven final photocyclization step, as detailed in the Supporting Information.

The MCBJ technique involves fixing the electrodes over a bending bead which provides high mechanical stability, as depicted in Figure 1(b). The electrodes consist of a gold wire that has been nanolithographed with a notch in the middle. A piezoelectric system applies a push to the wire which results in a controlled horizontal displacement, leading to elongation and finally the rupture at the notch. By cyclically bending and relaxing the electrodes, we create breaking and formation cycles, enabling precise control over the formation of metallic or molecular conductors. During this process, we measure the current flowing through the junction at a fixed bias voltage while stretching until it eventually breaks. We record the evolution of the conductance (in terms of G_0_ = 2*e*^2^/*h* with *e* being the elementary charge and *h* the Planck’s constant) as a function of the relative displacement between the leads forming a so-called breaking trace. An example of such a trace is depicted in Figure 1(c) while the rod is pushing until rupture. Additionally, we employ a logarithmic amplifier to achieve 9 orders of magnitude during data acquisition.^26^ By repeating the process thousands of times, we generate 1D histograms to depict the distribution of conductance values obtained. Furthermore, by overlaying the breaking traces, we create 2D histograms that present a density plot of the evolution of the most likely conductance values with the separation of the electrodes. These 2D/1D histograms as depicted in Figure 1(d), provide valuable insights for further analysis.

We have measured the electrical properties of the exo[7,9,11] and endo[11] molecules in dichloromethane (DCM) solution, considering both enantiomers. The measurements were performed with a concentration of 10 μM for exo[7] and 1 μM for the remaining molecules. For exo[7] we have recorded 2000 consecutive breaking traces while 10000 for the rest of the molecules. The use of low concentrations promotes single molecular bridges, although it may lead to a lower molecular yield. Figure 1(d) displays the collected data for the exo[11] helicenes in a 2*D*/1D histogram composition (as reference, Figure S3 of the Supporting Information depicts the 2D histograms of bare gold and the four molecules). Both histograms do not show clear peaks associated with single molecules because of the low molecular yield, which implies that the tunneling contribution dominates. An important observation in Figure 1(d) is the presence of a peak around 10^–6^ G_0_, which is an artifact introduced by the logarithmic amplifier. This peak value remains consistent across all the collected data and shows the expected decrease in conductance value^26^ as the bias voltage increases (see Supporting Information, Figure S4, which showcases the evolution of the peak).

In order to extract meaningful features from the recorded data sets and filter out the influence of the logarithmic amplifier artifact, we performed a two-step analysis. First, we utilized a neural network^27^ trained with a data set comprising breaking traces of bare gold and with molecules. This model, incorporating dropout layers, allowed us to classify the breaking traces and identify those with molecules bridging the electrodes. Once the classification is completed, we employ the k-means++ clustering algorithm,^28^ an unsupervised machine learning technique, to subtract the underlying molecular information. This algorithm partitions the data set into a predefined number of clusters. Through an iterative process, it identifies the most repeated values (conductance plateaus), so that traces with similar plateau structures converge into the same clusters. Consequently, this clustering analysis highlights the underlying molecular features within the predefined clusters. More details are provided in Supporting Information Figure S5, which shows a composition of 2*D*/1D histograms for all ten clusters obtained for the exo[11] molecule measured at 0.1 V. This includes the tunneling clusters, where no molecule was incorporated into the junction.

Turning to the clusters that contain molecular features, Figure 2 displays those for the data collected at 0.1 V for each molecule. Two main features can be obtained: First, the mean conductance value of the increased counts in the histogram (as derived from corresponding 1D histograms), and second, an estimated length of the conductance plateaus. To determine the mean conductance value (μ) from the 1D histogram, we represent the logarithmic data in a logarithmic binning scale. Gaussian functions are then fitted to the data using the formula . From this Gaussian fit, the mean conductance value is then extracted (see Table S1 in Supporting Information). In addition, we can define a lower conductance value within the cluster distribution as μ – 0.5σ. This value is used as criterion to limit the region of interest of the conductance plateaus. The length of each plateau is then computed with the start point fixed at the value of 0.3 G_0_ and the end point being the lower conductance limit defined as μ – 0.5σ. After collecting the lengths of the plateaus, a 1D histogram is constructed. This histogram is then used to estimate the overall plateau length for each cluster through Gaussian fitting. This plateau length has been found to correlate with the molecular length between binding sites in molecular junctions.^29,30^

**Figure 2:**
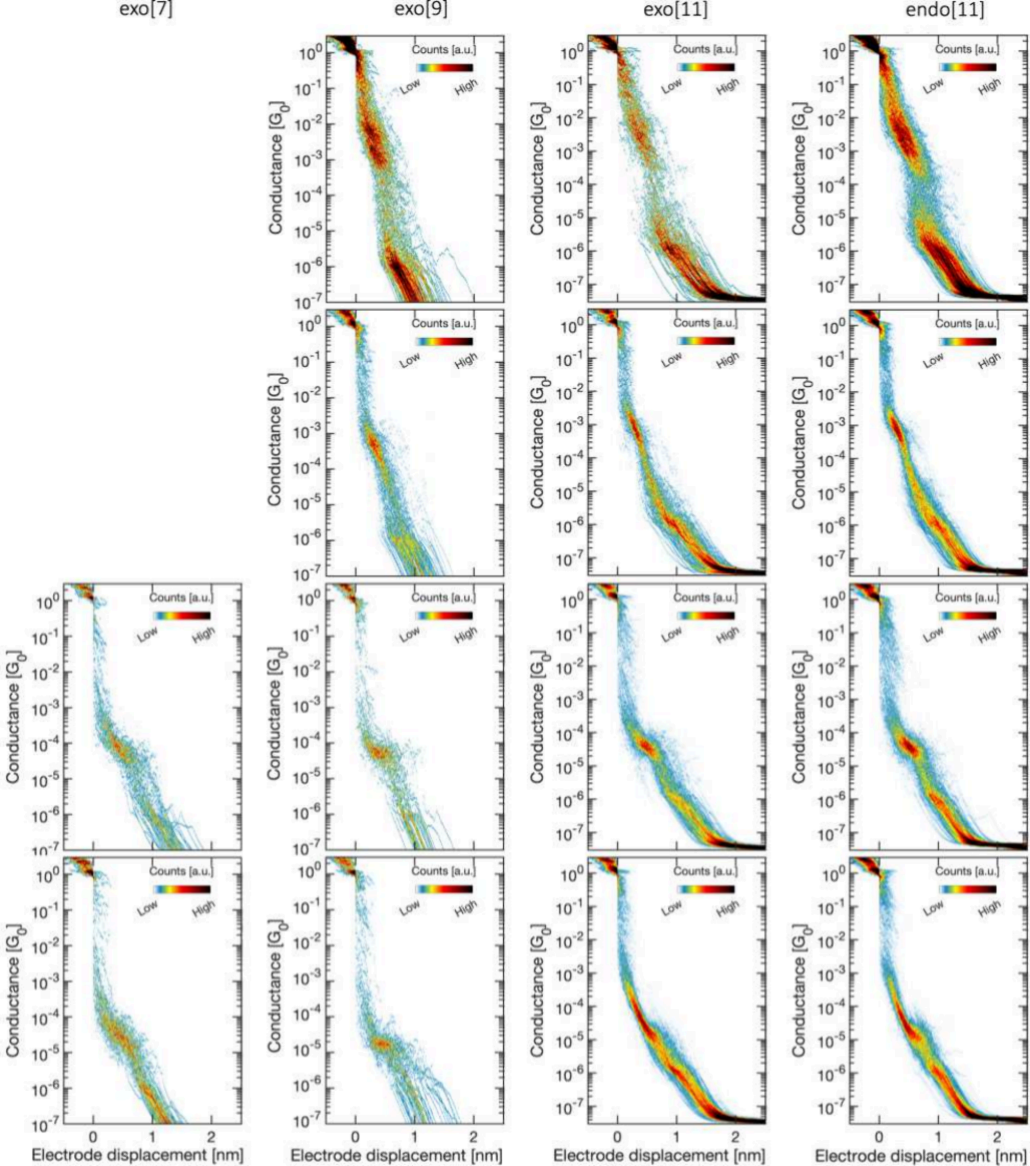
2D histograms of the main clusters of each helical molecule at 0.1 V. Each column in the histogram corresponds to a specific molecule, showcasing different clusters associated with distinct plateau structures ranging from 10^–5^ to 10^–3^ G_0_.

Figure 2 displays different clusters for each molecule taken at 0.1 V. The most prominent and frequently observed plateau structures converge into up to four assigned clusters. The mean conductance values fall within the range of 10^–3^ to 10^–5^ G_0_. The first row displays a wider distribution at a conductance around 10^–3^ G_0_, which may be indicative of at least two binding events. This may be a consequence of the helical structure of the molecule that results in slightly different contact configurations with similar conductance. By increasing the number of clusters when analyzing the data set might provide a more detailed local view, but we are interested in capturing the global picture. Furthermore, using a higher parameter for clustering may result in overly segmented distributions. To check for reproducibility and potential changes in conductance at higher voltage values,^31^ the measurements were repeated at different bias voltages. For exo[9, 11], the bias is ranged from 0.05 up to 0.35 V and up to 1 V for endo[11]. To perform statistical analysis, we recorded 10000 breaking traces, except for 0.7 and 1 V applied bias voltages, where 4000 and 2000 traces were obtained respectively, as it was difficult to find stable contacts, possibly due to thermal effects and electromigration.^32^ The [9] and [11] helicenes were selected based on the idea that longer molecules could achieve a greater variety in binding configurations.

The data sets measured at different bias voltages were simultaneously clustered, enabling the identification of common features as illustrated in Figures S7 and S8 in the Supporting Information. The fitted conductance values from each cluster measured are presented in Figure 3(a, b) as a function of bias voltage. The figure shows that the same clusters appear across the different bias voltages and that the conductance values are largely insensitive to changes in bias. This may indicate that the HOMO and LUMO are not close to the Fermi energy of the leads, i.e., the transmission function is nearly flat and far from the molecular orbitals. It is also noticeable that there is a slight change from panel (a) to (b) toward lower conductance values, only for the data points shown in blue. Since we are clustering data from different bias voltages, including those up to 1 V, this last result may be indicative of the stability of molecular junctions at high bias voltages. Additionally, the information subtracted from the 2D/1D histograms enables the determination of the averaged length of the plateaus in each cluster; the mean conductance values are plotted against these lengths in Figure 3(c, d). As observed, the blue data points in panels (a) and (b) exhibit similar lengths, despite showing slightly different conductance values. This may be a consequence of considering the same range of plateaus in both cases (panels (a, b)), indicating overall similar molecular events. Among the studied molecules, a consistent trend emerges with a characteristic decay of conductance with average plateau length.

**Figure 3:**
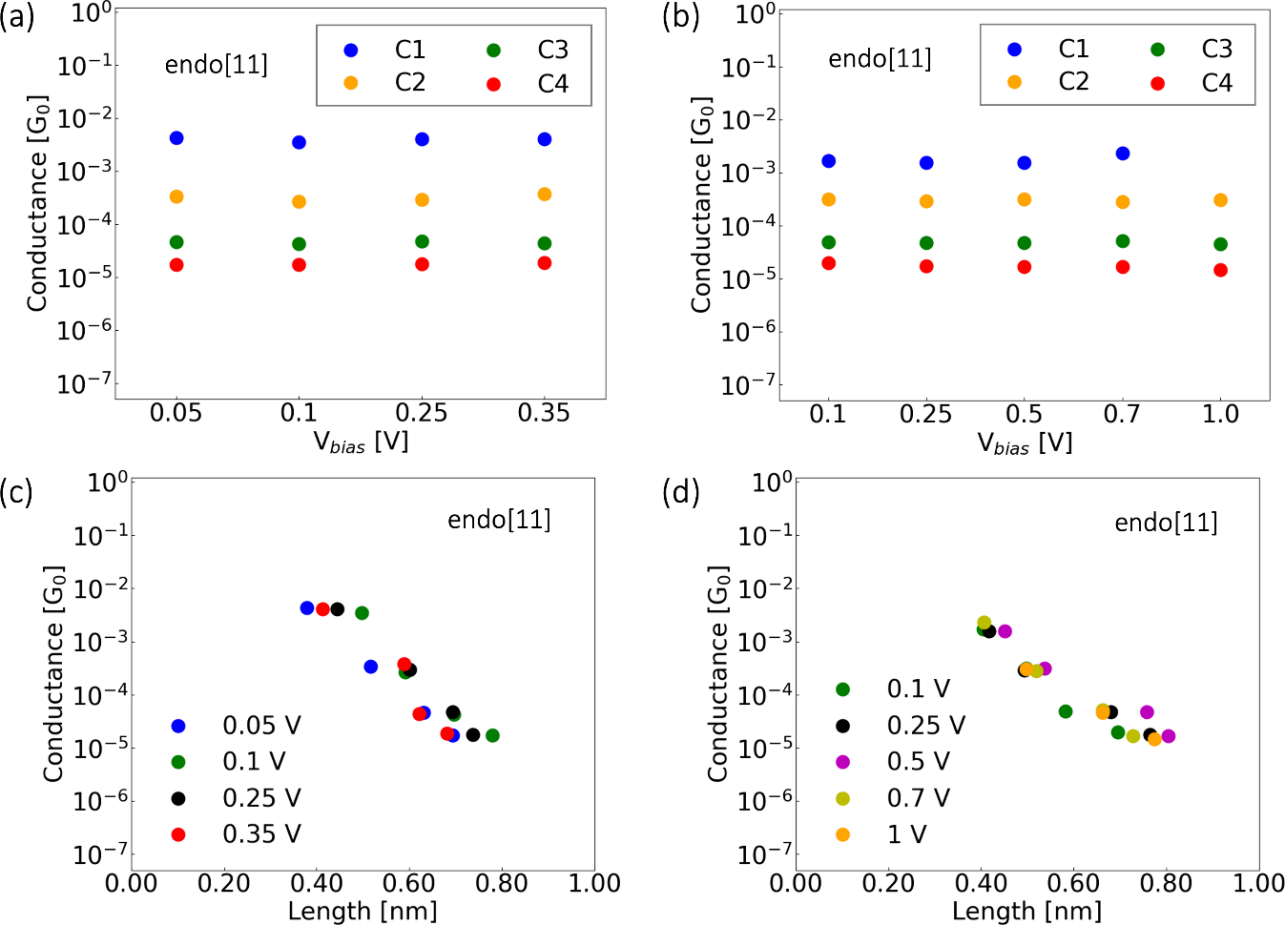
Mean conductance values of the four clusters (C1–C4) found for two data sets of endo[11] as a function of (a, b) the bias voltage and (c, d) the length. To improve clarity, common assignments in both (a) and (b) are color-coded identically, facilitating comparisons across various voltage levels. In contrast, panels (c, d) are color-coded based on the voltage data set. Panels (a, c) refer to one data set analyzed by using 10 clusters at low bias, while panels (b, d) represent another data set using 5 clusters for high bias voltages.

Figure 3(c) and (d) showcase the dependence with the length, indicating the possibility of contacting the molecules through different points and obtaining distinct conductance values. Furthermore, considering the repeatability and consistent trend observed, we calculated the average values of related clusters to determine the conductance value and the estimated length. One advantage of this approach is the large amount of data available for analysis. Additionally, when increasing the bias voltages, the molecular yield increases, possibly due to the electric field aiding the diffusion of molecules toward the tip. However, instabilities arise beyond 0.5 V, which poses a challenge for acquiring data. This drives us to compute the average for the available data set ranging from 0.05 to 0.35 V. The conductance value for exo[7] helicene, without additional measurements, was obtained from the measurements at 0.1 V. The averaged conductance values are summarized in Table 1.

**Table 1:** Mean Conductance Values for All Target Molecules and Different Clusters^a^

	exo[7]	exo[9]	exo[11]	endo[11]
G_C1_/G_0_		2.0 × 10^–3^	2.5 × 10^–3^	4.0 × 10^–3^
G_C2_/G_0_		1.7 × 10^–4^	2.7 × 10^–3^	3.2 × 10^–3^
G_C3_/G_0_	6.4 × 10^–5^	3.2 × 10^–5^	5.8 × 10^–5^	4.5 × 10^–5^
G_C4_/G_0_	2.8 × 10^–5^		2.4 × 10^–5^	1.8 × 10^–5^

a Conductance for exo[7] is obtained for *V*_bias_ = 0.1 V, while the mean values for the other molecules correspond to the average value among the different bias voltages up to 0.35 V. The clusters that show the most pronounced plateau structure are listed.

The averaged conductance vs relative displacement for the longer helicenes is presented in Figure 4(a). Here, the conductance decays with the length of the molecular plateau, which seems to follow an exponential decrease. This trend can be fitted by following the expression
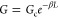
where *G* is the conductance, the inverse of *G*_c_ defines the contact resistance with left and right anchors,^33^ β is the decay constant related to the tunnelling barrier, and *L* is the length. This exponential decay can be understood as a sign for off-resonant transport,^34^ which significantly relies on the degree of localization and energy alignment of the molecular orbitals that mediate transport with the Fermi energy of the electrodes (sketched in panel (c) of Figure 4). It is important to stress that this approach is commonly carried out by measuring the conductance for different molecules as a function of the number of repeating units in the molecule. In that case, by measuring the conductance of a series of fully stretched molecules of different sizes (molecular units), information on β is obtained. Here, we use the approach to characterize charge transport along the molecule while it is stretched, as it will be contacted through different molecular positions, following the analysis on previous work on peptide chains.^35^

**Figure 4:**
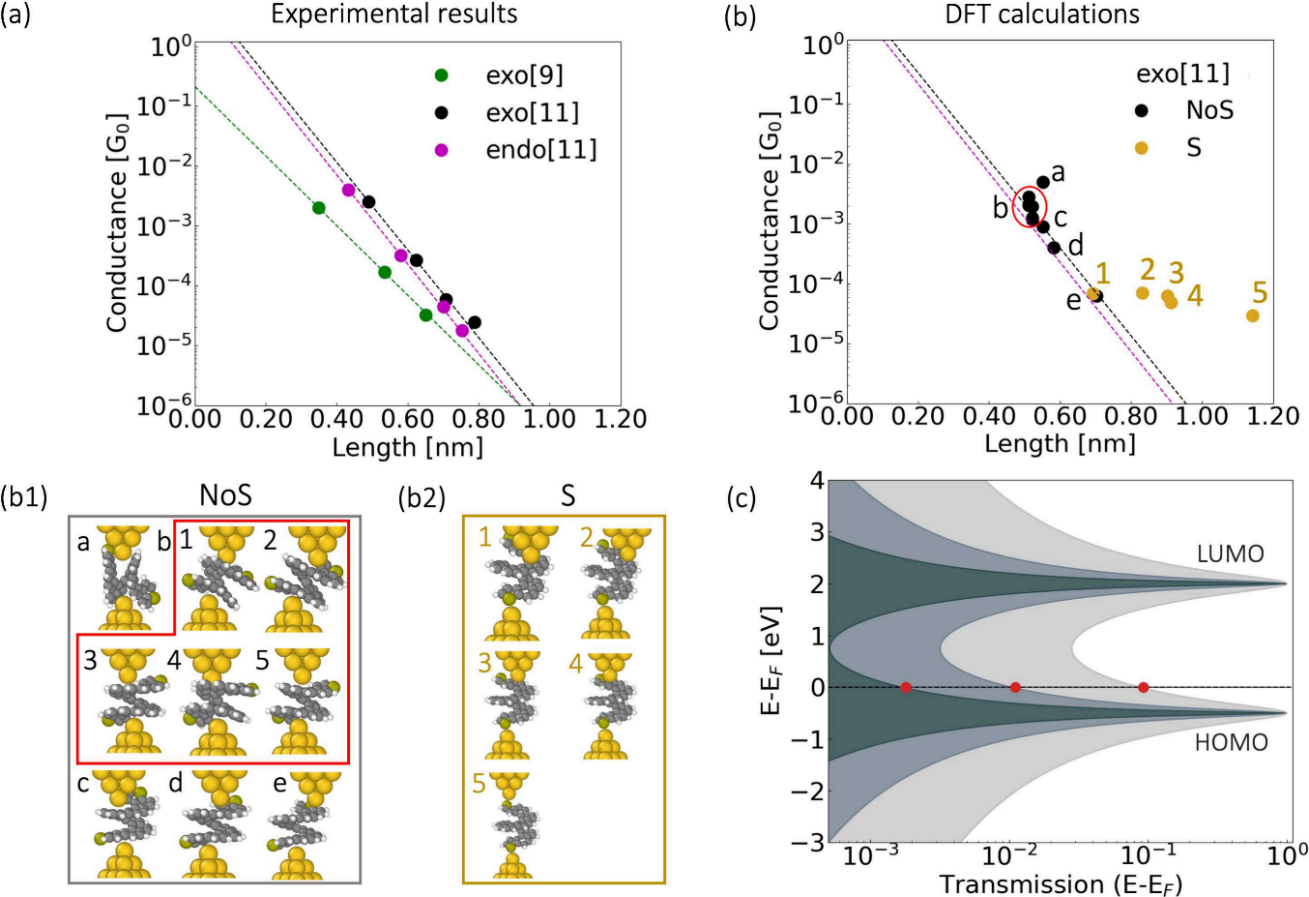
Correlation between molecular structure and electronic conductance. (a) Averaged experimental conductance as a function of the averaged length for the exo[9], endo[11], and exo[11] molecules. Dashed lines display the exponential fits. (b) Conductance data derived from DFT calculations, connected through the carbons, NoS, and through at least one of the sulfurs, S. Panels (b1) and (b2) depict the molecular structures associated with each data point in panel (b), illustrating the impact of molecular composition on conductance. (c) Schematic illustration depicting the off-resonant transport due to the energy misalignment between the molecular orbitals and the Fermi energy of the electrodes. The red dots mark the off-resonance electronic transport for the different conjugation strengths.

The β parameter gives information on the distance of the Fermi energy of the electrodes to the molecular levels. Several studies have reported small decay values for β related to conjugated systems such as oligophenylene and oligoacenes terminated in -S or -NC with β around 0.050 nm^–1^ or a molecular wire (DAD)_*n*_ chain with β = 0.021 nm^–1^.^36^ Smaller values for oligothiophenes were found for β = 0.01 nm^–1^.^37^ On the other hand, there have been reported values larger by an order of magnitude related to nonconjugated systems as there is also a dependence of the β parameter to the conjugation of molecule: alkanes with β = 7.5–10.0 nm^–1^,^38^ cysteamine-(n)glycinecysteine chains with β = 8.7 nm^–1^,^21^ triglycine with β = 9.7 nm^–1^,^39^ and peptide chains with β = 13.5–15.3 nm^–1^.^35^ In our case, for each helicene, as depicted in Figure 4, an exponential fit was applied to obtain the β parameter. As our molecules were designed to be conjugated as corroborated in our DFT calculations (see Figure S9), our β parameter shows the misalignment of the Fermi energy to the molecular orbital, i.e., off-resonant transport. The obtained β values are 13.40 ± 0.09 and 16.9 ± 0.2 nm^–1^ for the exo[9] and [11], respectively, and 17.18 ± 0.08 nm^–1^ for the endo[11]. Based on our DFT calculations from panel (b) of Figure 4, the molecules anchor predominantly through the conjugated backbone rather than through the thiophenes. This leads to no significant difference when comparing the exo and endo [11], meaning this motif does not play a significant role for the electronic transport in our experiments.

We employed *ab initio* calculations to support the experimental evidence and explore the influence of various molecular configurations on electronic transport. Density functional theory (DFT) was performed in order to optimize the geometries of the gold electrodes-molecule system, as displayed in Figure 4 (also refer to Figure S9 of the Supporting Information for the transmission curves of the optimized scenarios). To compute the conductance, a combination of Spin–Orbit Coupling-corrected DFT with nonequilibrium Green’s function (NEGF) was employed using the ANT.GAUSSIAN code.^40−42^ Moreover, the HSE06 functional was selected, which is extensively used in metal–organic systems.^43−46^ The Gaussian-type orbital basis sets employed in all electronic transport calculations in this manuscript are the same as those utilized by the authors in reference.^47^ In Figure 4, panel (b) showcases the computed conductance values vs the distance of the apex atoms of the gold electrodes, for all the optimized geometrical structures depicted in panels (b1) and (b2). The DFT conductances can be encompassed in two distinct situations: connection through the carbon atoms of the structure, noted as NoS, and connection through at least one sulfur atom, noted as S. Our first observation is regarding the impact of the molecular configurations on the conductance: connections through the helical structure (NoS) yield distinct signals which mainly depends on the distance and the geometry of the entire system, following what appears to be the observed experimental decay trends. However, once a sulfur atom participates in the transport, the conductance value seems to stabilize to values in the order of 10^–5^ G_0_, attributed to the barrier by the gold–sulfur contact.

Apparently, for the helicenes studied here, experimental results show predominantly up to four distinct conductance signatures, each associated with different anchor points on the molecule. Notably, there is a greater variety of contact configurations for longer molecules, leading to a common range of characteristic conductance plateaus for molecules of varying sizes. This scenario is supported by DFT calculations when the transport does not involve the sulfurs, which demonstrates that conductance is influenced by the binding geometry. We want to emphasize that now, thanks to the good agreement between experiments and theoretical calculations, we understand how these molecules adopt various geometric configurations relative to the electrodes and how these relate to their conductance characteristics.

Although the connection between helicenes and the electrodes, as well as the number of molecular units involved, remains unknown, the decay ratios obtained fall within the same range as those reported in nonconjugated systems. This suggests that transport is carried out by the electrons out of the resonance, resulting in lower conductance. This decay is supported qualitatively by the DFT calculations as shown in Figure 4.

The consistent behavior of the mean conductance value, regardless of changes in bias voltage, suggests that the measurements are taken outside the resonant transport regime. This indicates that the molecular energy levels are misaligned with respect to the Fermi level of the electrodes, and transport occurs at some energy within the molecular energy gap. In addition, conductance length dependence measurements along each molecule, further suggest that off-resonant transport is likely the primary mechanism of transport for helicenes under ambient conditions. Our analysis supports the use of the β decay analysis to get information on the details of the transport mechanism in molecular electronics. Furthermore, this result implies a limitation when detecting the CISS effect in these helicenes due to the low conductance values obtained and the misalignments of the energy levels of the system. This suggests the need to improve charge transport in these systems, which may involve modifying the molecular structure design or selecting different electrodes for conducting transport experiments.

## Supporting Information Available

Synthesis of dithia[*n*]helicenes mentioned in the text, details on the clustering algorithm and the used parameters, and description of the functional and basis used in the density functional calculations (PDF)

## References

[ref1] Cuevas J. C.; Scheer E. Molecular Electronics: An Introduction to Theory and Experiment; World Scientific Series in Nanoscience and Nanotechnology; World Scientific, 2010.

[ref2] Evers F.; Korytár R.; Tewari S.; van Ruitenbeek J. M. Advances and Challenges in Single-Molecule Electron Transport. Rev. Mod. Phys. 2020, 92, 035001 10.1103/RevModPhys.92.035001.

[ref3] Evers F.; Aharony A.; Bar-Gill N.; Entin-Wohlman O.; Hedegård P.; Hod O.; Jelinek P.; Kamieniarz G.; Lemeshko M.; Michaeli K.; Mujica V.; et al. Theory of Chirality Induced Spin Selectivity: Progress and Challenges. Adv. Mater. 2022, 34, 2106629 10.1002/adma.202106629. 35064943

[ref4] Göhler B.; Hamelbeck V.; Markus T.; Kettner M.; Hanne G.; Vager Z.; Naaman R.; Zacharias H. Spin Selectivity in Electron Transmission Through Self-Assembled Monolayers of Double-Stranded DNA. Science 2011, 331, 894–897. 10.1126/science.1199339. 21330541

[ref5] Yang X.; van der Wal C. H.; van Wees B. J. Detecting Chirality in Two-Terminal Electronic Nanodevices. Nano Lett. 2020, 20, 6148–6154. 10.1021/acs.nanolett.0c02417. 32672980 PMC7458476

[ref6] Dednam W.; García-Blázquez M. A.; Zotti L. A.; Lombardi E. B.; Sabater C.; Pakdel S.; Palacios J. J. A Group-Theoretic Approach to the Origin of Chirality-Induced Spin-Selectivity in Nonmagnetic Molecular Junctions. ACS Nano 2023, 17, 6452–6465. 10.1021/acsnano.2c11410. 36947721 PMC10100547

[ref7] García-Blázquez M. A.; Dednam W.; Palacios J. J. Nonequilibrium Magneto-Conductance as a Manifestation of Spin Filtering in Chiral Nanojunctions. J. Phys. Chem. Lett. 2023, 14, 7931–7939. 10.1021/acs.jpclett.3c01922. 37646507 PMC10494227

[ref8] Naskar S.; Mujica V.; Herrmann C. Chiral-Induced Spin Selectivity and Non-equilibrium Spin Accumulation in Molecules and Interfaces: A First-Principles Study. J. Phys. Chem. Lett. 2023, 14, 694–701. 10.1021/acs.jpclett.2c03747. 36638217

[ref9] Kiran V.; Mathew S. P.; Cohen S. R.; Hernández Delgado I.; Lacour J.; Naaman R. Helicenes—A new Class of Organic Spin Filter. Adv. Mater. 2016, 28, 1957–1962. 10.1002/adma.201504725. 26742997

[ref10] Matxain J. M.; Ugalde J. M.; Mujica V.; Allec S. I.; Wong B. M.; Casanova D. Chirality Induced Spin Selectivity of Photoexcited Electrons in Carbon-Sulfur [n] Helicenes. ChemPhotoChem. 2019, 3, 770–777. 10.1002/cptc.201900128.

[ref11] Giaconi N.; Poggini L.; Lupi M.; Briganti M.; Kumar A.; Das T. K.; Sorrentino A. L.; Viglianisi C.; Menichetti S.; Naaman R.; et al. Efficient Spin-Selective Electron Transport at Low Voltages of Thia-Bridged Triarylamine Hetero [4] helicenes Chemisorbed Monolayer. ACS Nano 2023, 17, 15189–15198. 10.1021/acsnano.3c04878. 37493644 PMC10416567

[ref12] Kettner M.; Maslyuk V. V.; Nürenberg D.; Seibel J.; Gutierrez R.; Cuniberti G.; Ernst K.-H.; Zacharias H. Chirality-Dependent Electron Spin Filtering by Molecular Monolayers of Helicenes. J. Phys. Chem. Lett. 2018, 9, 2025–2030. 10.1021/acs.jpclett.8b00208. 29618210

[ref13] Safari M. R.; Matthes F.; Schneider C. M.; Ernst K.-H.; Bürgler D. E. Spin-Selective Electron Transport Through Single Chiral Molecules. Small 2024, 20, 2308233 10.1002/smll.202308233. 38050945

[ref14] Safari M. R.; Matthes F.; Caciuc V.; Atodiresei N.; Schneider C. M.; Ernst K.-H.; Bürgler D. E. Enantioselective Adsorption on Magnetic Surfaces. Adv. Mater. 2024, 36, 2308666 10.1002/adma.202308666. 38153192

[ref15] Baciu B. C.; de Ara T.; Sabater C.; Untiedt C.; Guijarro A. Helical Nanostructures for Organic Electronics: the Role of Topological Sulfur in Ad hoc Synthesized Dithia [7] helicenes Studied in the Solid State and on a Gold Surface. Nanoscale Adv. 2020, 2, 1921–1926. 10.1039/D0NA00045K. 36132536 PMC9417725

[ref16] Baciu B. C.; Bronk P. J.; de Ara T.; Rodriguez R.; Morgante P.; Vanthuyne N.; Sabater C.; Untiedt C.; Autschbach J.; Crassous J.; Guijarro A. Dithia[9]helicenes: Molecular Design, Surface Imaging, and Circularly Polarized Luminescence with Enhanced Dissymmetry Factors. J. Mater. Chem. C 2022, 10, 14306–14318. 10.1039/D2TC02910C.

[ref17] Venkataraman L.; Klare J.; Tam I.; Nuckolls C.; Hybertsen M.; Steigerwald M. Single-Molecule Circuits with Well-Defined Molecular Conductance. Nano Lett. 2006, 6, 458–62. 10.1021/nl052373+. 16522042

[ref18] Reed M. A.; Zhou C.; Muller C. J.; Burgin T. P.; Tour J. M. Conductance of a Molecular Junction. Science 1997, 278, 252–254. 10.1126/science.278.5336.252.

[ref19] Li C.; Pobelov I.; Wandlowski T.; Bagrets A.; Arnold A.; Evers F. Charge Transport in Single Au — Alkanedithiol — Au Junctions: Coordination Geometries and Conformational Degrees of Freedom. J. Am. Chem. Soc. 2008, 130, 318–326. 10.1021/ja0762386. 18076172

[ref20] Zheng J.; Liu J.; Zhuo Y.; Li R.; Jin X.; Yang Y.; Chen Z.-B.; Shi J.; Xiao Z.; Hong W.; Tian Z.-q. Electrical and SERS Detection of Disulfide-Mediated Dimerization in Single-Molecule Benzene-1,4-dithiol Junctions. Chem. Sci. 2018, 9, 5033–5038. 10.1039/C8SC00727F. 29938032 PMC5994741

[ref21] Xiao; Xu; Tao Conductance Titration of Single-Peptide Molecules. J. Am. Chem. Soc. 2004, 126, 5370–5371. 10.1021/ja049469a. 15113203

[ref22] Kim Y.; Hellmuth T. J.; Burkle M.; Pauly F.; Scheer E. Characteristics of Amine-Ended and Thiol-Ended Alkane Single-Molecule Junctions Revealed by Inelastic Electron Tunneling Spectroscopy. ACS Nano 2011, 5, 4104–4111. 10.1021/nn200759s. 21506567

[ref23] Kaliginedi V.; Rudnev A. V.; Moreno-García P.; Baghernejad M.; Huang C.; Hong W.; Wandlowski T. Promising Anchoring Groups for Single-Molecule Conductance Measurements. Phys. Chem. Chem. Phys. 2014, 16, 23529–23539. 10.1039/C4CP03605K. 25285778

[ref24] Treboux G.; Lapstun P.; Wu Z.; Silverbrook K. Electronic Conductance of Helicenes. Chem. Phys. Lett. 1999, 301, 493–497. 10.1016/S0009-2614(99)00085-8.

[ref25] Vacek J.; Chocholoušová J. V.; Stará I. G.; Starý I.; Dubi Y. Mechanical Tuning of Conductance and Thermopower in Helicene Molecular Junctions. Nanoscale 2015, 7, 8793–8802. 10.1039/C5NR01297J. 25905658

[ref26] Ornago L. Complexity of Electron Transport in Nanoscale Molecular Junctions. Doctoral thesis, Delft University of Technology, 2023; TU Delft QN/van der Zant Lab.

[ref27] van Veen F.; Ornago L.; van der Zant H. S.; El Abbassi M. Generalized Neural Network Approach for Separation of Molecular Breaking Traces. J. Mater. Chem. C 2023, 11, 15564–15570. 10.1039/D3TC02346J.

[ref28] Cabosart D.; El Abbassi M.; Stefani D.; Frisenda R.; Calame M.; van der Zant H. S. J.; Perrin M. A Reference-Free Clustering Method for the Analysis of Molecular Break-Junction Measurements. Appl. Phys. Lett. 2019, 114, 143102 10.1063/1.5089198.

[ref29] Untiedt C.; Yanson A. I.; Grande R.; Rubio-Bollinger G.; Agraït N.; Vieira S.; van Ruitenbeek J. Calibration of the Length of a Chain of Single Gold Atoms. Phys. Rev. B 2002, 66, 085418 10.1103/PhysRevB.66.085418.

[ref30] Martinez-Garcia A.; de Ara T.; Pastor-Amat L.; Untiedt C.; Lombardi E.; Dednam W.; Sabater C. Unraveling the Interplay between Quantum Transport and Geometrical Conformations in Monocyclic Hydrocarbons’ Molecular Junctions. J. Phys. Chem. C 2023, 127, 23303–23311. 10.1021/acs.jpcc.3c05393. PMC10861133 38352239

[ref31] Koch M.; Ample F.; Joachim C.; Grill L. Voltage-Dependent Conductance of a Single Graphene Nanoribbon. Nat. Nanotechnol. 2012, 7, 713–717. 10.1038/nnano.2012.169. 23064554

[ref32] Sabater C.; Untiedt C.; van Ruitenbeek J. M. Evidence for Non-Conservative Current-Induced Forces in the Breaking of Au and Pt Atomic Chains. Beilstein J. Nanotechnol. 2015, 6, 2338–2344. 10.3762/bjnano.6.241. 26734525 PMC4685917

[ref33] Engelkes V. B.; Beebe J. M.; Frisbie C. D. Length-Dependent Transport in Molecular Junctions Based on SAMs of Alkanethiols and Alkanedithiols: Effect of Metal Work Function and Applied Bias on Tunneling Efficiency and Contact Resistance. J. Am. Chem. Soc. 2004, 126, 14287–14296. 10.1021/ja046274u. 15506797

[ref34] Moth-Poulsen K.; Bjørnholm T. Molecular Electronics with Single Molecules in Solid-State Devices. Nat. Nanotechnol. 2009, 4, 551–556. 10.1038/nnano.2009.176. 19734925

[ref35] Stefani D.; Guo C.; Ornago L.; Cabosart D.; El Abbassi M.; Sheves M.; Cahen D.; van der Zant H. S. J. Conformation-Dependent Charge Transport Through Short Peptides. Nanoscale 2021, 13, 3002–3009. 10.1039/D0NR08556A. 33508063

[ref36] Nacci C.; Ample F.; Bleger D.; Hecht S.; Joachim C.; Grill L. Conductance of a Single Flexible Molecular Wire Composed of Alternating Donor and Acceptor Units. Nat. Commun. 2015, 6, 7397 10.1038/ncomms8397. 26145188 PMC4507002

[ref37] Yamada R.; Kumazawa H.; Noutoshi T.; Tanaka S.; Tada H. Electrical Conductance of Oligothiophene Molecular Wires. Nano Lett. 2008, 8, 1237–1240. 10.1021/nl0732023. 18311936

[ref38] van Veen F. H.; Ornago L.; van der Zant H. S. J.; El Abbassi M. Benchmark Study of Alkane Molecular Chains. J. Phys. Chem. C 2022, 126, 8801–8806. 10.1021/acs.jpcc.1c09684.

[ref39] Park Y. S.; Whalley A. C.; Kamenetska M.; Steigerwald M. L.; Hybertsen M. S.; Nuckolls C.; Venkataraman L. Contact Chemistry and Single-Molecule Conductance: A Comparison of Phosphines, Methyl Sulfides, and Amines. J. Am. Chem. Soc. 2007, 129, 15768–15769. 10.1021/ja0773857. 18052282

[ref40] Palacios J. J.; Pérez-Jiménez A. J.; Louis E.; Vergés J. A. Fullerene-Based Molecular Nanobridges: A First-Principles Study. Phys. Rev. B 2001, 64, 115411 10.1103/PhysRevB.64.115411.

[ref41] Palacios J. J.; Pérez-Jiménez A. J.; Louis E.; SanFabián E.; Vergés J. A. First-Principles Approach to Electrical Transport in Atomic-Scale Nanostructures. Phys. Rev. B 2002, 66, 035322 10.1103/PhysRevB.66.035322.

[ref42] Dednam W.; Zotti L. A.; Palacios J. J. ANT.Gaussian. GitHub, 2024. https://github.com/juanjosepalacios/ANT.Gaussian (accessed 2023-02-15).

[ref43] Heyd J.; Scuseria G. E.; Ernzerhof M. Hybrid Functionals Based on a Screened Coulomb Potential. J. Chem. Phys. 2003, 118, 8207–8215. 10.1063/1.1564060.

[ref44] Heyd J.; Scuseria G. E. Efficient Hybrid Density Functional Calculations in Solids: Assessment of the Heyd–Scuseria–Ernzerhof Screened Coulomb Hybrid Functional. J. Chem. Phys. 2004, 121, 1187–1192. 10.1063/1.1760074. 15260659

[ref45] Camarasa-Gómez M.; Ramasubramaniam A.; Neaton J. B.; Kronik L. Transferable Screened Range-Separated Hybrid Functionals for Electronic and Optical Properties of Van der Waals Materials. Phys. Rev. Mater. 2023, 7, 104001 10.1103/PhysRevMaterials.7.104001.

[ref46] Yin W.-J.; Tan H.-J.; Ding P.-J.; Wen B.; Li X.-B.; Teobaldi G.; Liu L.-M. Recent Advances in Low-Dimensional Janus Materials: Theoretical and Simulation Perspectives. Mater. Adv. 2021, 2, 7543–7558. 10.1039/D1MA00660F.

[ref47] Dednam W.; García-Blázquez M. A.; Zotti L. A.; Lombardi E. B.; Sabater C.; Pakdel S.; Palacios J. J. A Group-Theoretic Approach to the Origin of Chirality-Induced Spin-Selectivity in Nonmagnetic Molecular Junctions. ACS Nano 2023, 17, 6452–6465. 10.1021/acsnano.2c11410. 36947721 PMC10100547

