# Supplementary material for: Evidence of an Off-Resonant Electronic Transport Mechanism in Helicenes
Source: J Phys Chem Lett. 2024 Aug 7;15(32):8343–50. doi: 10.1021/acs.jpclett.4c01425 (PMC11331518; doi:10.1021/acs.jpclett.4c01425)
Supplement: Supplementary file 1 — jz4c01425_si_001.pdf [file jz4c01425_si_001.pdf]

Evidence of an Off-resonant Electronic Transport Mechanism in Helicenes

T. de Ara,[†] C. Hsu,[‡] A. Martinez-Garcia,[†] B. C. Baciu,[¶] P. J. Bronk,[¶] L. Ornago,[‡] S. van der Poel,[‡] E. B. Lombardi,[§] A. Guijarro,[¶] C. Sabater,[†] C. Untiedt,[*,†] and H. S. J. van der Zant[*,‡]

†*Departamento de Física Aplicada and Instituto Universitario de Materiales de Alicante (IUMA), Universidad de Alicante, Campus de San Vicente del Raspeig, E-03690 Alicante, Spain.*

‡*Department of Quantum Nanoscience, Delft University of Technology, Delft 2628CJ, The Netherlands.*

¶*Departamento de Química Orgánica and Instituto Universitario de Síntesis Orgánica, Universidad de Alicante, Campus de San Vicente del Raspeig, E-03690, Alicante, Spain*

§*Department of Physics, Florida Science Campus, University of South Africa, Florida Park, Johannesburg 1710, South Africa*

E-mail: untiedt@ua.es; h.s.j.vanderzant@tudelft.nl

Supplementary Information

Synthesis of dithia[n]helicenes: exo[7], exo[9], exo[11] and endo[11] dithiahelicenes

Fragments **1**, **2** and **3** in the synthetic scheme below are described in our previous works.[1,2] The details and characterization of bis-stilbenic precursors **4** and **5** and final dithiahelicenes exo[11] (**6**) and endo[11] (**7**) follows.

Bis-stilbenic intermediates **4** and **5** needed to obtain the final dithia[11]helicenes were prepared by a Suzuki-Miyaura coupling between 3,6-dibromophenanthrene (**3**) as the central fragment,[1] and the corresponding isomer of vinyl boronic pinacol esters (**1** or **2**).[2] These boronic esters bear the exo-/endo- structural features in the type of fusion of their terminal thiophene ring, and so is eventually transferred to the final helical compound. Yields of products are given in parenthesis (Figure S1).

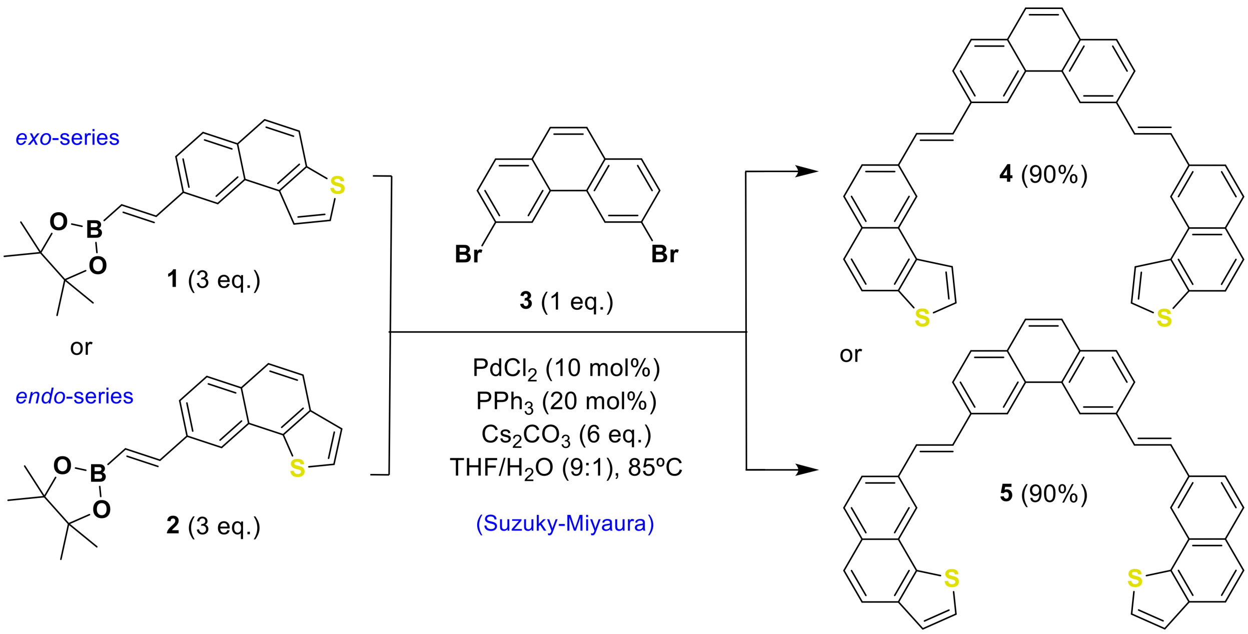

Figure S1: Synthesis of bis-stilbenic precursors 4 and 5 for exo- and endo-dithia[11]helicenes

Compounds **4** and **5** are the photochemical precursors of the new dithia[11]helicenes, namely exo[11] and endo[11], respectively. The key photochemical step is a double Mal-

lory reaction. It was performed employing a 365 nm light source from LED plates, using iodine as oxidant and an excess of 1,2-butyleneoxide as an acid quencher. The reactions were performed under inert atmosphere (Ar) (Figure S2). The characterization of all new compounds, i.e. both bis-stilbenic precursors and dithia[11]helicenes is described in the Supporting Information.

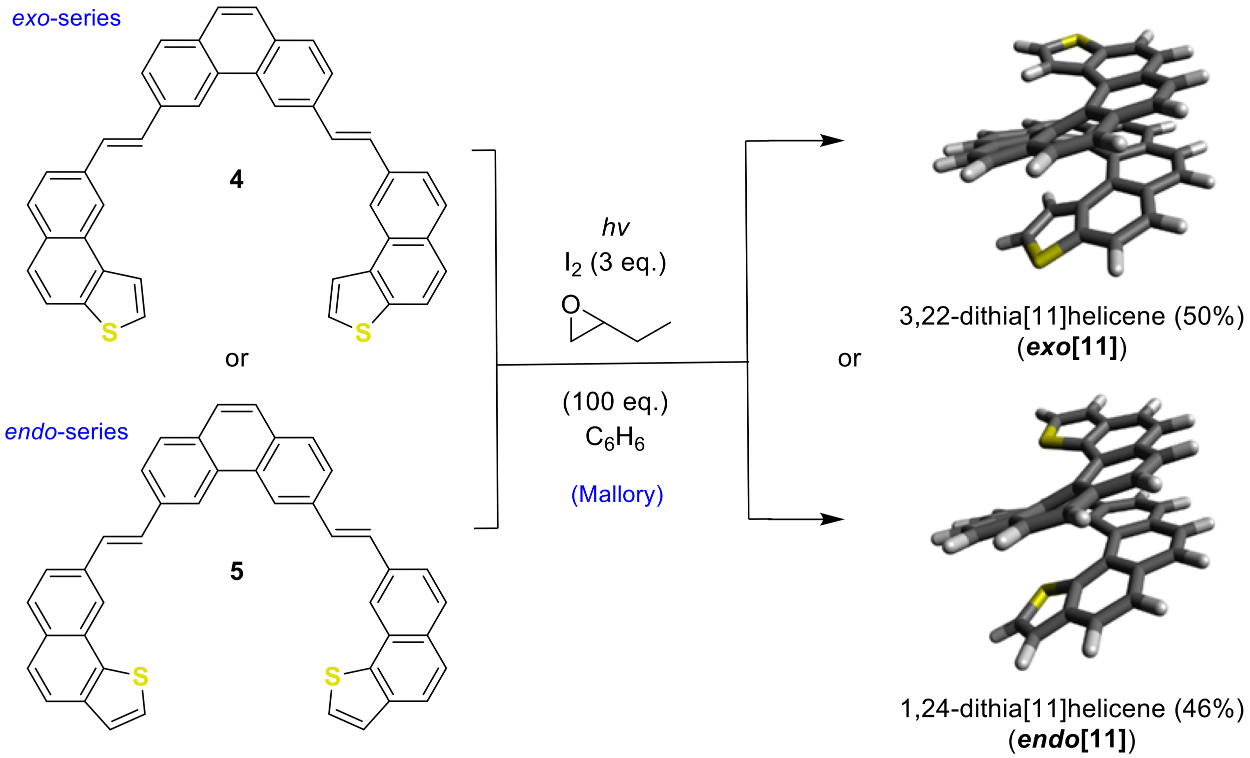

Figure S2: Final oxidative photocyclization step obtaining exo- and endo-dithia[11]helicenes.

Synthesis and characterization of bis-stilbenic precursors 4 and 5, exo-dithia[11]helicene (6) and endo-dithia[11]helicene (7)

3,6-bis((E)-2-(naphtho[2,1-b]tiophen-8-yl)vinyl)phenanthrene (4)

The synthesis of this compound was carried out by a Suzuki-Miyaura reaction under the following conditions: In an over-dried pressure tube $PdCl_2$ (4.4 mg; 0.025 mmol; 0.10 eq.), PPh_3 (13.1 mg; 0.05 mmol; 0.20 eq.), Cs_2CO_3 (488.73 mg; 1.5 mmol; 6 eq.), 3,6-dibromophenanthrene (84.10 mg; 0.25 mmol; 1 eq.) were added. The tube was sealed with a

septum and after three cycles of vacuum/argon, (E)-4,4,5,5-tetramethyl-2-(2-(naphtho[2,1-b]tiophen-8-yl)vinyl)-1,3,2-dioxoborolane (**1**) (252.11 mg, 0.75 mmol, 3 eq.) dissolved in 1.8 mL of THF were added, followed by 0.2 mL of H_2O, both of them via syringe. After that, the tube was closed and heated in an oil bath at 85℃ for 20 hours. An insoluble solid in suspension was observed in the tube. This insoluble yellow solid was filtered, washed with H_2O and CH_2Cl_2 and used without further purification.

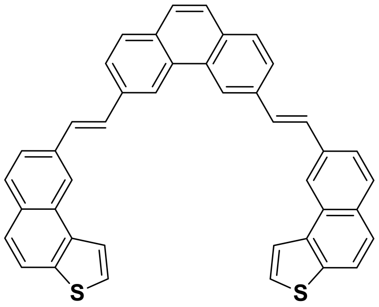

Yellow solid in 90% yield; MS (EI, DIP) m/z: 596.1 (M^++2, 20), 595.1 (M^++1, 48), 594.1 (M^+, 100), 545.1 (28), 544.1 (71), 408.1 (13), 358.1 (14), 297.1 (20), 272.0 (15), 197.0 (11). IR (neat) ν_{max}: 3062, 3020, 1616, 1373, 1192, 1153, 953, 876, 833, 710, 663 cm^{-1}.

3,6-bis((E)-2-(naphtho[1,2-b]tiophen-8-yl)vinyl)phenanthrene (5)

This compound was prepared following the previous procedure, but using (E)-4,4,5,5-tetramethyl-2-(2-(naphtho[1,2-b]tiophen-8-yl)vinyl)-1,3,2-dioxoborolane (**2**) as starting reagent.

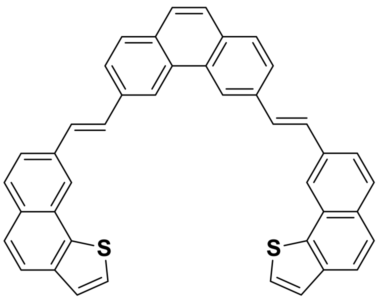

Yellow solid in 90% yield; MS (EI, DIP) m/z 596.1 (M^++2, 20), 595.1 (M^++1, 47), 594.1 (M^+, 100), 546.1 (16), 545.1 (30), 544.1 (70), 408.1 (13), 384.1 (8), 358.1 (13), 297.1 (26),

272.0 (17), 197.0 (14). IR (neat) ν_{max}: 2974, 2885, 1612, 1311, 1261, 1088, 1045, 949, 876, 833, 702 cm^{-1}.

3,22-dithia[11]helicene (exo-dithia[11]helicene) (6)

In an oven-dried 250mL Schlenk tube 3,6-bis((E)-2-(naphtho[2,1-b]tiophen-8-yl)vinyl)phenanthrene (**4**) (17.84 mg, 0.03 mmol, 1 eq.) was added, followed by 200 mL of benzene. The mixture was stirred and heated a little bit to dissolve the reagent. After that, iodine (22.84 mg, 0.09 mmol, 3 eq.) and 1,2-butyleneoxide (0.3 mL, 3 mmol, 100 eq.) were added. The solution was bubbled with Ar for 15 minutes and then two 265 nm LEDs plates of 50 W each were turned on. With the light on, the solution was bubbled with Ar for 15 more minutes and then the tube was closed. The mixture was irradiated with LEDs overnight. After the reaction was completed, it was washed with aqueous $NaHSO_3$, then H_2O, dried with anhydrous magnesium sulphate, filtered and the solvent evaporated under reduced pressure (15 Torr). The residue was purified by column chromatography on silica gel (hexane-CH_2Cl_2 8:2) to obtain a yellow solid. The product was recrystallized in CH_2Cl_2 to obtain yellow crystals.

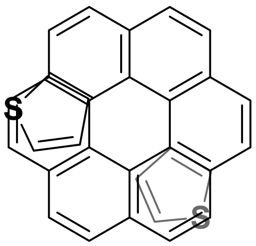

Yellow crystals in 50% yield; R_f= 0.45 (hexane-CH_2Cl_2 6:4); ^{1}H-NMR ($CDCl_3$, 400MHz): δ= 7.61 (s, 2H), 7.47 (dd, J= 8.5, 0.8 Hz, 2H), 7.38 (d, J= 8.2 Hz, 2H), 7.34 (d, J= 8.4 Hz, 2H), 7.27 (d, J= 8.3 Hz, 2H), 7.23 (d, J= 8.3 Hz, 2H), 7.22 (d, J= 8.2 Hz, 2H), 7.22 (s, 4H), 6.37 (dd, J= 5.6, 0.4 Hz, 2H), 5.81 (dd, J= 5.6, 0.8 Hz, 2H) ppm. ^{13}C-NMR ($CDCl_3$, 101 MHz): δ=137.44 (-C-, 2C), 133.97 (-C-, 2C), 131.95 (-C-, 2C), 131.10 (-CH-, 2C), 130.04 (-C-, 2C), 127.24 (-C-, 2C), 126.76 (-CH-, 2C), 126.46 (-CH-, 2H), 126.18 (-CH-, 2C), 126.14 (-CH-, 2C), 125.63 (-CH-, 2C), 125.54 (-CH-, 2C), 124.62 (-CH-, 2C), 124.57 (-C-, 2C),

124.46 (-C-, 2C), 123.51 (-C-, 2C), 122.72 (-CH-, 2C), 121.59 (-CH-, 2C), 121.08 (-C-, 2C), 119.85 (-CH-, 2C), 118.91 (-C-, 2C) ppm. MS (EI, DIP) m/z: 592.2 (M^++2, 20), 591.2 (M^++1, 49), 590.2 (M^+, 100), 306.1 (12), 295.1 (26), 282.1 (11), 277.0 (18), 276.1 (15), 263.1 (10). IR (neat) ν_{max}: 3039, 1315, 1292, 1138, 1092, 949, 891, 833, 783, 714, 683 cm^{-1}.

1,24-dithia[11]helicene (endo-dithia[11]helicene) (7)

This compound was prepared following the previous procedure, but using 3,6-bis((E)-2-(naphtho[1,2-b]tiophen-8-yl)vinyl)phenanthrene (**6**) as starting reagent. The residue was purified by column chromatography on silica gel (hexane-CH_2Cl_2 8:2) to obtain a yellow solid. The product was recrystallized in CH_2Cl_2 to obtain yellow crystals.

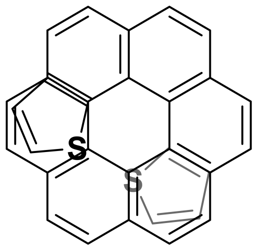

Yellow crystals in 46% yield; R_f= 0.52 (hexane-CH_2Cl_2 6:4); 1H-NMR ($CDCl_3$, 300MHz): δ= 7.72 (s, 2H), 7.46 (d, J= 8.2 Hz, 2H), 7.44 (d, J= 8.4 Hz, 2H), 7.39 (d, J= 8.4 Hz, 2H), 7.30 (d, J=8.4 Hz, 2H), 7.24 (d, J= 8.2 Hz, 2H), 7.23 (d, J= 8.3 Hz, 2H), 7.21 (s, 4H), 6.83 (d, J= 5.4 Hz, 2H), 6.63 (d, J= 5.4 Hz, 2H) ppm. ^{13}C-NMR ($CDCl_3$, 101 MHz): δ=137.20 (-C-, 2C), 135.32 (-C-, 2C), 132.86 (-C-, 2C), 131.39 (-C-, 2C), 130.88 (-C-, 2C), 130.05 (-C-, 2C), 127.33 (-C-, 2C), 127.03 (-CH-, 2H), 127.00 (-CH-, 2C), 126.29 (-CH-, 2C), 126.19 (-CH-, 2C), 125.92 (-CH-, 2C), 125.69 (-CH-, 2C), 125.61 (-CH-, 2C), 124.69 (-CH-, 2C), 124.37 (-C-, 2C), 123.12 (-CH-, 2C), 122.73 (-C-, 2C), 122.72 (-C-, 2C), 121.56 (-CH-, 2C), 120.92 (-CH-, 2C) ppm. MS (EI, DIP) m/z: 592.2 (M^++2, 19), 591.1 (M^++1, 45), 590.2 (M^+, 100), 295.1 (23), 277.2 (11), 270.7 (12), 245.1 (7). IR (neat) ν_{max}: 3039, 1300, 1265, 949, 833, 752, 717, 679 cm^{-1}.

3,22-dithia[11]helicene

^{1}H-NMR ($CDCl_3$, 400 MHz)

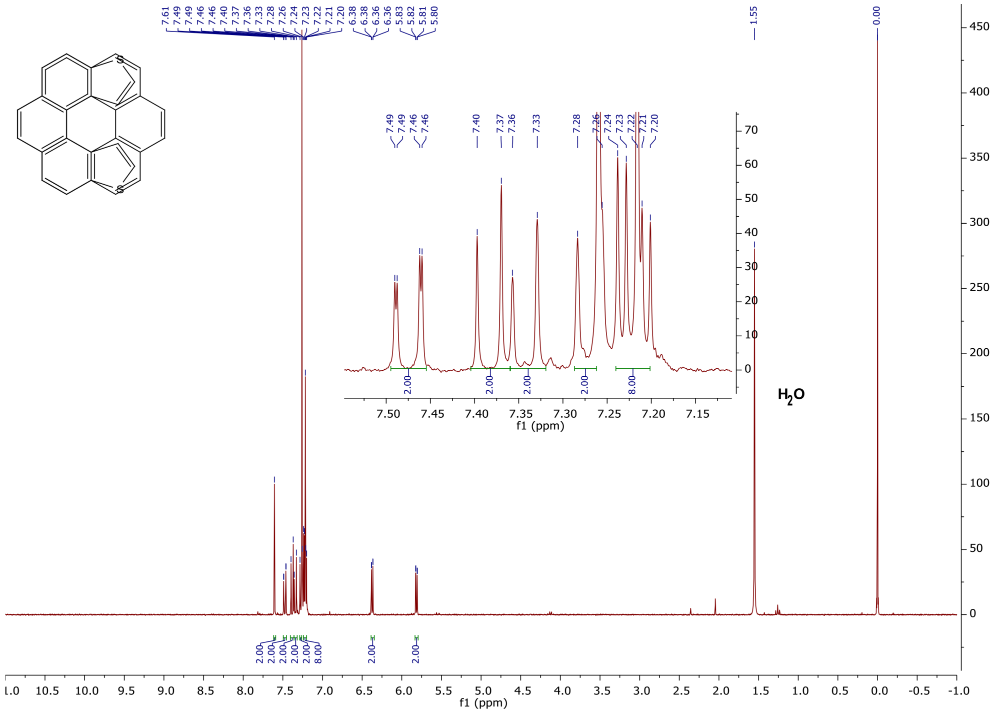

^{13}C-NMR ($CDCl_3$, 101 MHz)

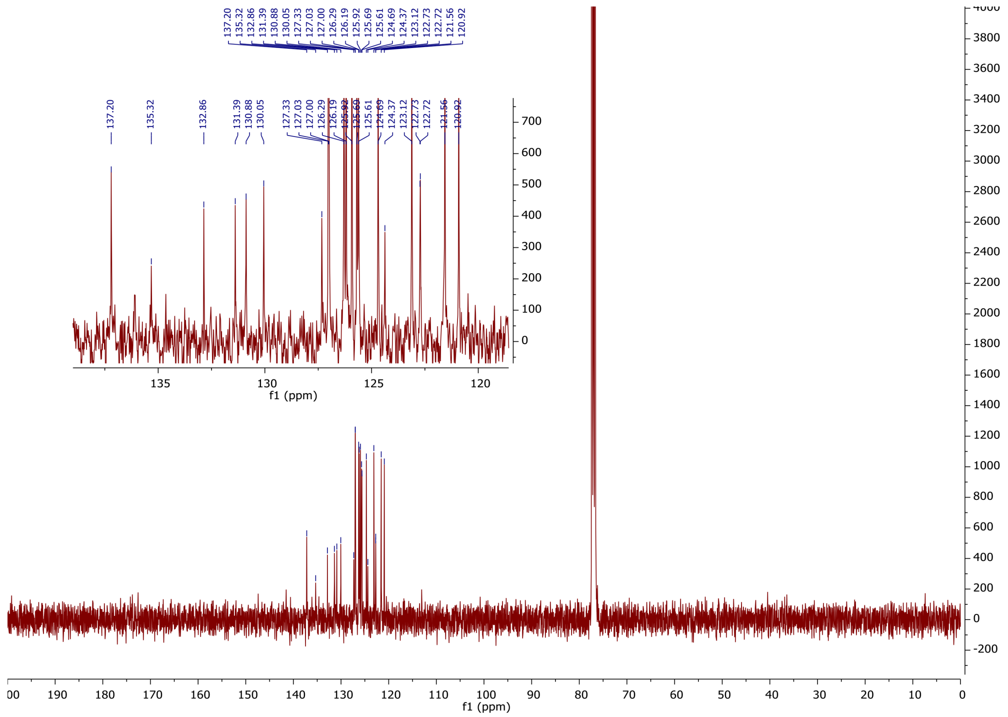

1,24-dithia[11]helicene

^{1}H-NMR ($CDCl_3$, 300 MHz)

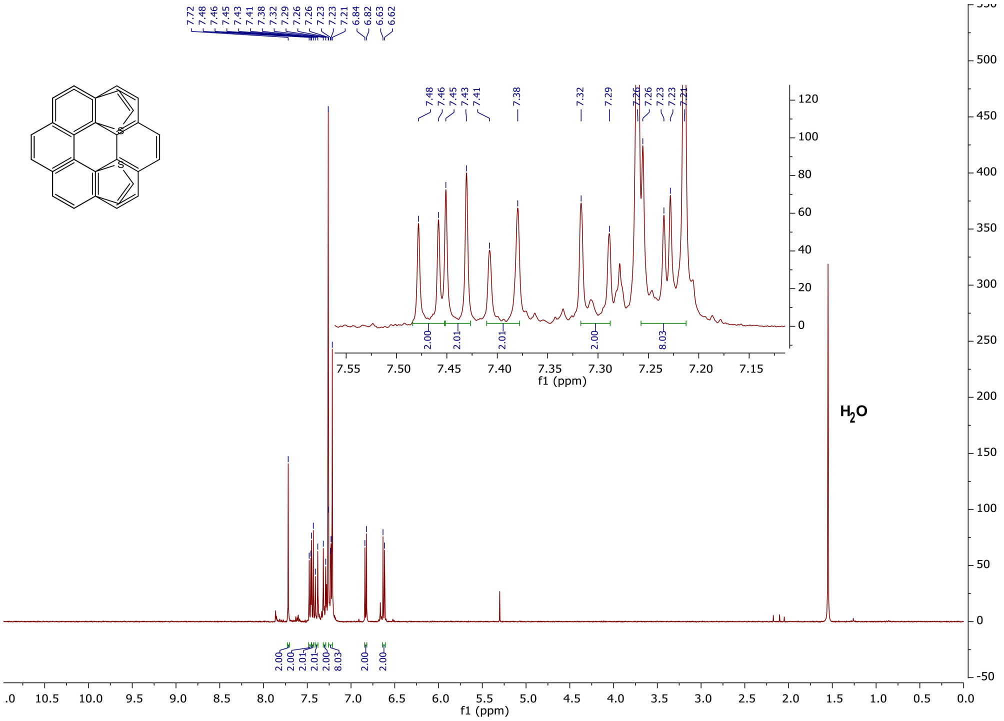

^{13}C-NMR ($CDCl_3$, 101 MHz)

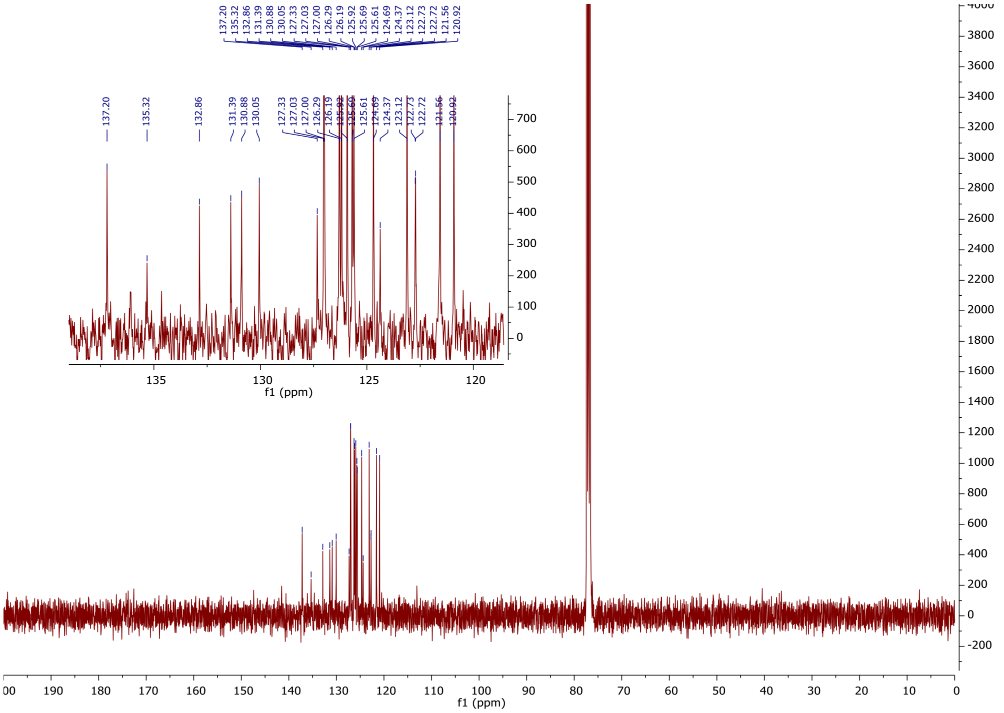

2D histograms of the raw data of all helicenes measured at 0.1 V

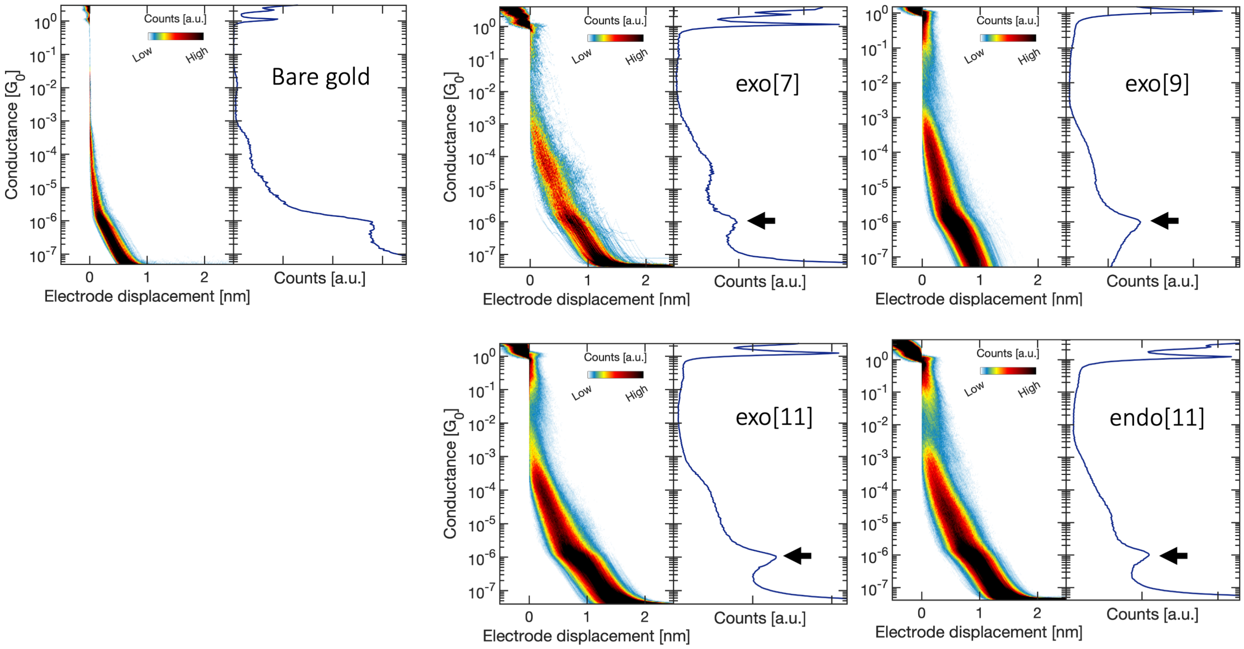

Figure S3: 2D histograms collecting all the raw data measured using MCBJ of bare gold and gold with helicenes.

Evolution of the peak related to the log-amplifier with the bias voltage

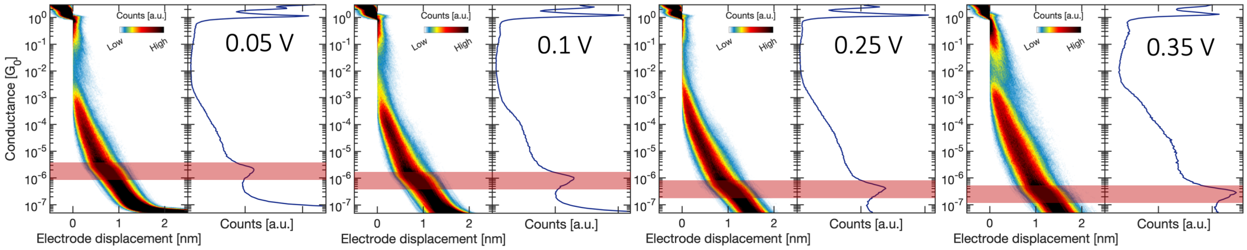

Figure S4: Raw data obtained via MCBJ of exo[11]helicenes at different bias voltages. The peak related to the amplifier artifact goes down in value when increasing the bias voltage. The evolution is highlighted using a red-shaded area.

Details for running the clustering technique

The input for the clustering considers a 2D histogram of each trace built by a matrix of 30x30 bins which is flattened by concatenating its rows. For this work, the algorithm focuses on

the region between 10^{-2} to 10^{-5} G_0, where molecular signatures typically appear, avoiding the region where the tunneling is the main contribution. The image is also built considering the range from 0 to 2 nm in displacement. Additionally, the 1D histogram is appended as input with 100 bins and limits -2 to -5 $\log(G/G_0)$.

This input offers the details required for clustering the breaking traces into the predefined number of clusters. For the analysis of the data presented, the classification was performed using 3 clusters for the exo[7], and 10 clusters for the exo[9,11] and endo[11]. The output for the exo[11] is presented in Figure S5. Each 2D histogram is presented along with the corresponding 1D histogram. The Blue shaded area shows the Gaussian fit. Table S1 collects the mean conductance values obtained.

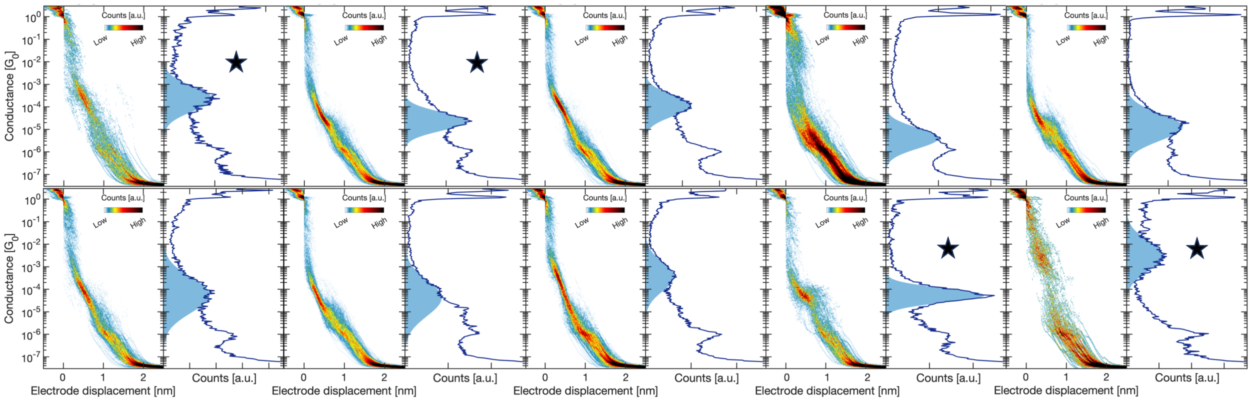

Figure S5: 2D/1D histograms of all the clusters obtained for the case of exo[11] helicenes measured at 0.1V. Marked clusters are the ones selected as examples in Figure 2 of the main text.

Table S1: Logarithmic fitted conductance values (μ) and logarithmic deviations (σ) of the main assignments of the helicenes.

exo[7]	exo[9]	exo[11]	endo[11]
	-2.5 ± 0.2	-2.4 ± 0.2	-2.5 ± 0.3
	-3.31 ± 0.09	-3.15 ± 0.08	-3.14 ± 0.03
-4.19 ± 0.08	-4.3 ± 0.3	-4.4 ± 0.2	-4.4 ± 0.2
-4.56 ± 0.07	-4.8 ± 0.4	-4.83 ± 0.01	-4.82 ± 0.02

From the Gaussian fit, a length histogram can be built as to obtain an estimation of the most repeated plateau length of each cluster. An example of the composition of a 2D/1D

histograms is shown in Figure S6 along the length histogram built for that cluster.

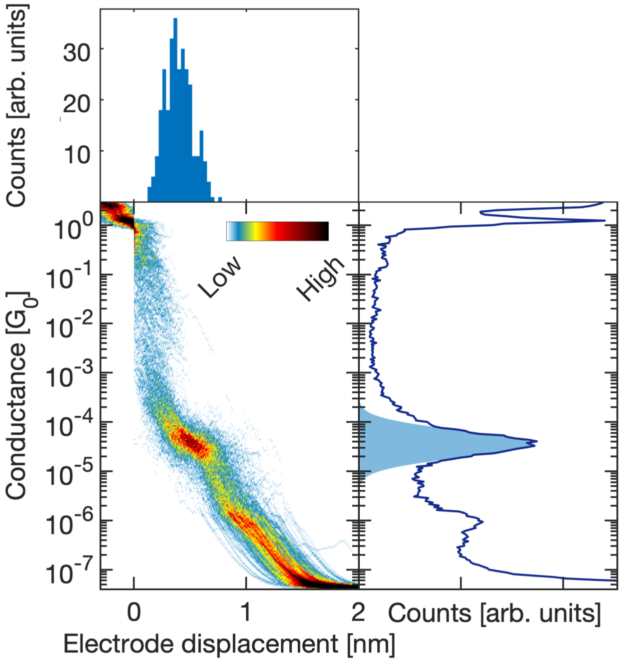

Figure S6: 2D/1D histogram of the conductance along 1D histogram of the length.

Cluster evolution with the bias voltage

As reference, Figures S7 and S8 display the common clusters across different bias voltages.

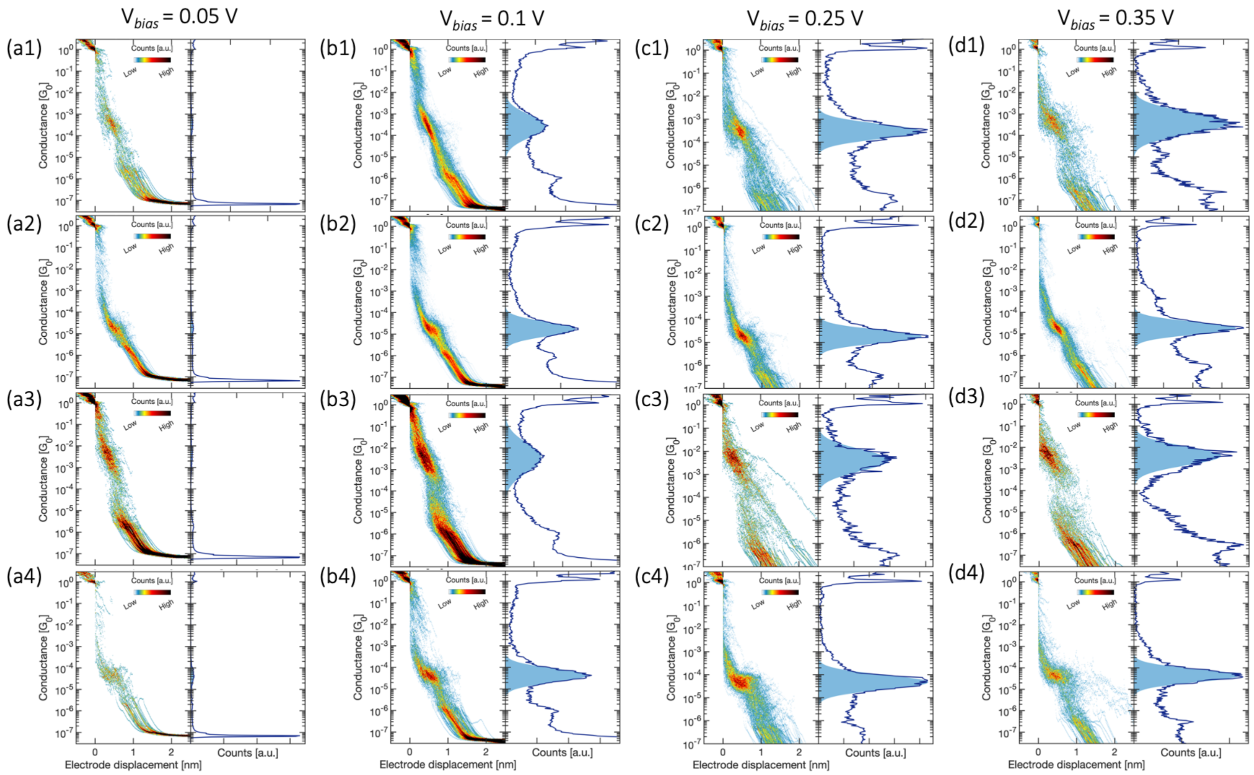

Figure S7: Examples to observe the cluster evolution with the bias voltage for the endo[11] using 10 clusters.

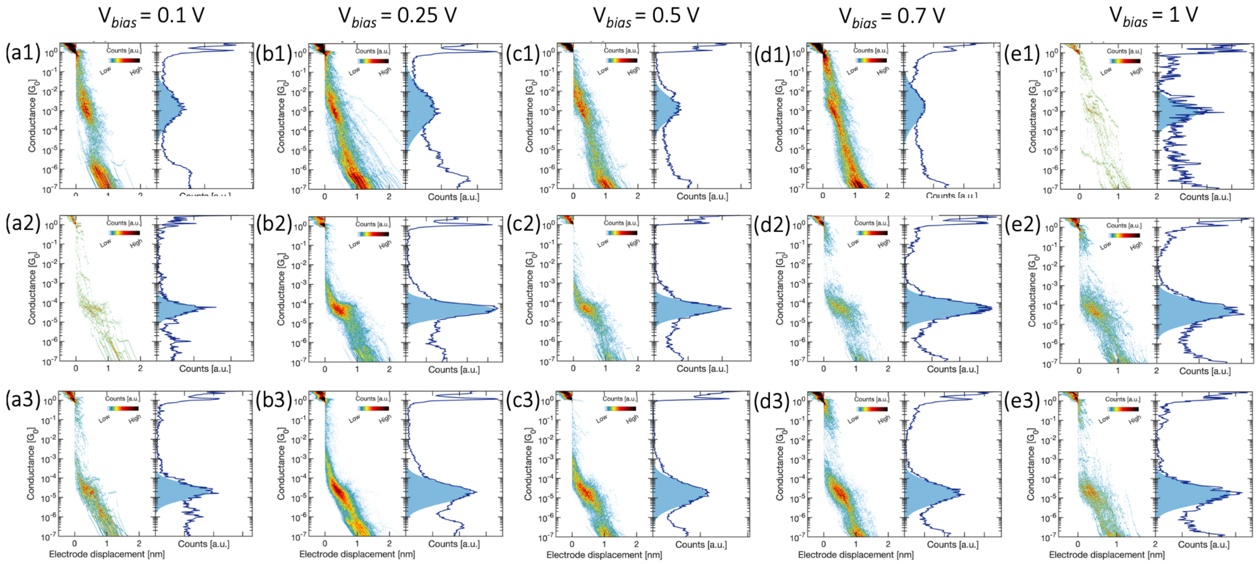

Figure S8: Examples to observe the cluster evolution with the high bias voltage for the endo[11] using 10 clusters.

Density functional theory details and transmission curves

Before conducting the conductance calculations, a geometry optimization is performed for the electrode-molecule system. For our calculations, HSE06 functional was employed as it is widely used in metal-organic systems.[3–6] The conductance calculations were performed using ANT.GAUSSIAN code,[7–9] which is built upon GAUSSIAN09. For specific details, refer to the main text and supplementary materials of reference.[10]

The computed curves of transmission, in terms of conductance (G_0) as a function of energy, are shown in a vertically stacked graph in Figure S9(a). The upper panel corresponds to molecules that are linked by at least one sulfur atom (labeled 1-5). In contrast, the middle and bottom panels show scenarios where the molecules are connected without any sulfur (labeled a-e and b1-b5). The latter ones are marked by a red circle in Figure 4(b) of the main text, as they correspond to a cluster of points of configurations with similar conductance values. Additionally, panel (b) illustrates the legends corresponding to the aforementioned panel. It can be observed that the three grouped curves exhibit similar shapes. In all cases, although the HOMO is close to the Fermi energy, it is out of resonance.

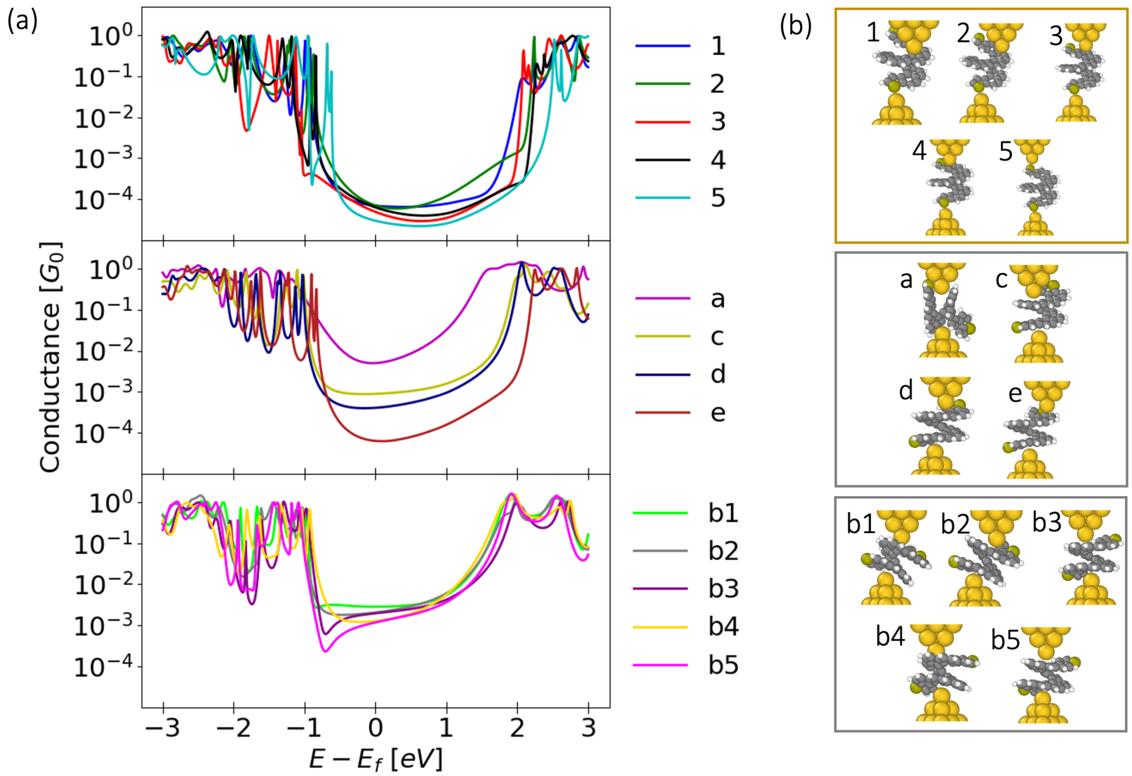

Figure S9: Panel (a) displays conductance curves obtained as a function of energy. The upper section features molecules linked by sulfur, and the middle and bottom showcase scenarios without sulfur connections. The curves exhibit similar patterns within each group. Panel (b) provides illustrations of the legends from the previous panel.

References

(1) Baciu, B. C.; de Ara, T.; Sabater, C.; Untiedt, C.; Guijarro, A. Helical Nanostructures for Organic Electronics: the Role of Topological Sulfur in Ad hoc Synthesized Dithia [7] helicenes Studied in the Solid State and on a Gold Surface. *Nanoscale Adv.* **2020**, *2*, 1921–1926.

(2) Baciu, B. C.; Bronk, P. J.; de Ara, T.; Rodriguez, R.; Morgante, P.; Vanthuyne, N.; Sabater, C.; Untiedt, C.; Autschbach, J.; Crassous, J. et al. Dithia[9]helicenes: Molecular Design, Surface Imaging, and Circularly Polarized Luminescence with Enhanced Dissymmetry Factors. *J. Mater. Chem. C* **2022**, *10*, 14306–14318.

(3) Heyd, J.; Scuseria, G. E.; Ernzerhof, M. Hybrid Functionals Based on a Screened Coulomb Potential. *J. Chem. Phys.* **2003**, *118*, 8207–8215.

(4) Heyd, J.; Scuseria, G. E. Efficient Hybrid Density Functional Calculations in Solids: Assessment of the Heyd–Scuseria–Ernzerhof Screened Coulomb Hybrid Functional. *J. Chem. Phys.* **2004**, *121*, 1187–1192.

(5) Camarasa-Gómez, M.; Ramasubramaniam, A.; Neaton, J. B.; Kronik, L. Transferable Screened Range-Separated Hybrid Functionals for Electronic and Optical Properties of Van der Waals Materials. *Phys. Rev. Mater.* **2023**, *7*, 104001.

(6) Yin, W.-J.; Tan, H.-J.; Ding, P.-J.; Wen, B.; Li, X.-B.; Teobaldi, G.; Liu, L.-M. Recent Advances in Low-Dimensional Janus Materials: Theoretical and Simulation Perspectives. *Mater. Adv.* **2021**, *2*, 7543–7558.

(7) Palacios, J. J.; Pérez-Jiménez, A. J.; Louis, E.; Vergés, J. A. Fullerene-Based Molecular Nanobridges: A First-Principles Study. *Phys. Rev. B* **2001**, *64*, 115411.

(8) Palacios, J. J.; Pérez-Jiménez, A. J.; Louis, E.; SanFabián, E.; Vergés, J. A. First-Principles Approach to Electrical Transport in Atomic-Scale Nanostructures. *Phys. Rev. B* **2002**, *66*, 035322.

(9) Dednam, W.; Zotti, L. A.; Palacios, J. J. Computer Code ANT.Gaussian, with SOC Corrections. Available from `https://github.com/juanjosepalacios/ANT.Gaussian`, Date of access: 15-Feb-2023.

(10) Dednam, W.; García-Blázquez, M. A.; Zotti, L. A.; Lombardi, E. B.; Sabater, C.; Pakdel, S.; Palacios, J. J. A Group-Theoretic Approach to the Origin of Chirality-Induced Spin-Selectivity in Nonmagnetic Molecular Junctions. *ACS Nano* **2023**, *17*, 6452–6465.
